# Childcare needs as a barrier to healthcare among women in a safety-net health system

**DOI:** 10.1186/s12889-024-19125-1

**Published:** 2024-06-17

**Authors:** Priyanka Gaur, Anisha P. Ganguly, Madyson Kuo, Robert Martin, Kristin S. Alvarez, Kavita P. Bhavan, Kimberly A. Kho

**Affiliations:** 1 Department of Gynecology and Obstetrics, The Johns Hopkins University School of Medicine, Baltimore, MD United States of America; 2 Center of Innovation and Value at Parkland, Parkland Health, Dallas, TX United States of America; 3 Department of Internal Medicine, University of Texas Southwestern Medical Center, Dallas, TX United States of America; 4 Department of Obstetrics and Gynecology, Methodist Health System, Dallas, TX United States of America; 5 Department of Obstetrics and Gynecology, University of Texas Southwestern Medical Center, Dallas, TX United States of America; 6 Parkland Health Center for Innovation and Value at Parkland, 5200 Harry Hines Blvd, Dallas, TX 75235 USA

**Keywords:** Childcare, Social determinant of health, Access to healthcare, Missed appointments, Deferred care

## Abstract

**Background:**

Childcare needs are an understudied social determinant of health. The effect of childcare needs on access to healthcare must be understood to inform health system interventions and policy reform. This study sought to characterize childcare needs, access to childcare, and prior experience with navigating childcare needs in healthcare settings among women in a safety-net population.

**Methods:**

We conducted a cross-sectional study of patient-reported survey data collected in-person between April and October 2019. Surveys were administered in waiting rooms of ambulatory services in a large, urban safety-net health system in Dallas, Texas. Survey respondents were derived from a random convenience sample of women waiting for outpatient appointments. Participants were screened for having children under the age of 13 and/or childcare responsibilities for inclusion in the sample. Outcomes of interest included self-reported delayed or missed care, reasons for delayed or missed care, perceived difficulty in accessing childcare, prior methods for managing childcare during healthcare appointments, and prior experience with childcare centers.

**Results:**

Among the 336 respondents (96.7% response rate), 121 (36.0%) reported delaying or missing a mean 3.7 appointments/year. Among women with delayed or missed care, 54.5% reported childcare barriers as the primary reason for deferral of care, greater than transportation (33%) or insurance (25%) barriers. Respondents rated childcare access as more difficult than healthcare access. Delayed or missed care due to childcare was more common among White (68.8%) and Black (55.0%) women compared to Hispanic women (34.3%). Common methods of navigating childcare needs during scheduled appointments included bringing children to appointments (69.1%) and re-scheduling or missing the scheduled appointment (43.0%). 40.6% of patients reported leaving an appointment before completion due to childcare needs.

**Conclusions:**

Childcare needs are a leading barrier to healthcare among women accessing care in safety-net settings. Unmet childcare needs result in deferral of care, which may impact health outcomes. Childcare access is perceived as more challenging than healthcare access itself. Health system and policy interventions are needed to address childcare as a social determinant of health.

**Supplementary Information:**

The online version contains supplementary material available at 10.1186/s12889-024-19125-1.

## Introduction

Social determinants of health (SDOH) such as access to transportation [1, 2], sick leave from work [3], and insurance [4] are widely recognized as factors that impact patients’ ability to access health services [5–7]. While both men and women experience barriers accessing healthcare, studies have demonstrated that women are more likely to delay or forego healthcare when faced with financial and structural barriers to accessing healthcare [8]. In an analysis by the Commonwealth Fund, more than half of women reported postponing preventative services, skipping recommended tests or treatments, or missing prescriptions because of costs, compared to 39% of men [9]. The 2017 Kaiser Women’s Health Survey illustrated that despite the national expansion of health insurance coverage under the Affordable Care Act, cost-related and logistical barriers like time off, transportation, and childcare present unique barriers to care among women [10]. Such barriers are exacerbated among women from lower income backgrounds, including those with Medicaid coverage or without insurance [10]. 

Costs of healthcare have been shown to disproportionately affect women, as women on average have fewer financial resources and are more likely to experience poverty than men [11]. Furthermore, women experience unique logistical barriers related to their role as caretakers; traditional gender roles task women with significant unpaid labor in caring for their families and children [12–14]. Women from lower income backgrounds particularly face increased complexity in navigating childcare among competing financial needs [15]. Such convergence of co-existing SDOH was unmasked and amplified during the COVID-19 pandemic, during which women struggled to balance work, financial strain, and childcare needs [16]. Pandemic conditions have underscored the need for childcare policy reform to increase investment in access to childcare for low income families [17, 18]. Federally funded childcare programs such as Head Start and Early Head Start and childcare subsidies aim to support low income families through childcare support and school readiness [19], but access to childcare support remains constrained by limited federal investment and a depleted childcare workforce [17, 19, 20] Current discourse on childcare policy centers on the economic impact of access to childcare, while the impact of childcare needs on health needs remains underrecognized.

While logistical barriers to healthcare like transportation and insurance have been previously explored in the SDOH literature [21–24], the role of childcare needs on healthcare access remains less understood. Prior studies have suggested that childcare needs pose a barrier to women’s engagement in prenatal care [25, 26], cancer screening and treatment [27–29], and substance use disorder treatment [30, 31]. However, childcare needs are often elicited alongside other health-related social needs (HRSN), rather than through dedicated screening tools, and additional investigation is needed to understand unmet childcare needs as a unique, discrete HRSN. Furthermore, policy discourse on childcare needs has centered on the economic impact and workplace costs caused by the shortage of affordable childcare [32], whereas implications of unmet childcare needs on healthcare access and impact to the health and wellbeing of caregivers remain under-explored. This study sought to characterize childcare needs among women in a large safety-net health system population and examine how childcare needs impacted access to healthcare.

## Methods

We conducted a needs assessment of women accessing ambulatory services in a large safety-net health system in Dallas County, Texas. This study was reviewed and considered exempt by the University of Texas Southwestern Medical Center Institutional Review Board. The quantitative needs assessment was comprised of categorical survey questions and Likert scale assessments adapted from survey questions administered in the 2017 KFF Women’s Health Survey and prior work from the Commonwealth Fund [10, 33]. Surveys were administered using a script in English and Spanish to a convenience sample of patients in clinic waiting rooms between April and October 2019. Survey questions were validated in a pilot phase of cognitive testing between January and April 2019 among 100 respondents with guidance from a biostatistician with experience in survey design and the use of qualified bilingual language services. A priori sample size for the study phase was selected as 300 completed surveys, and surveys were incorporated into analysis if greater than 70% completed.

Clinics sampled included adult primary and subspecialty care, obstetrics and gynecology, and surgical and medical oncology. Inclusion criteria for participation included women with children present or women who appeared of reproductive age per the interviewer’s assessment. Eligible patients were further screened by the following: “Do you have any children living at home under the age of 13?” and “Do you have or share childcare responsibilities for the children under age 13 in your home?” The age threshold of 13 for children was used due to our health system’s institutional policy requiring supervision of children under 13 and prohibiting any unattended children younger than 13 in clinical areas; this institutional policy is informed by Texas Department of Family and Protective Services recommendations for child supervision [34]. Patients answering affirmatively to both questions were informed about the study and verbally consented to complete the survey which averaged 15 min in length.

The interviewer recorded responses on an encrypted tablet. The interviewer recorded patient arrival and departure times, number of patients, and number of children in clinic waiting rooms daily. The complete survey instrument is available as Supplement 1. Our primary areas of interest were patient-reported volume of missed healthcare appointments, estimated length of delays in care (e.g., less than 1 week, 1 week to 1 month, 1 month to 6 months, more than 6 months), barriers to healthcare appointments (e.g., transportation, work leave, coverage) and patient perception of ease accessing medical care and arranging childcare measured by Likert Scale assessment (e.g., very difficult, moderately difficult, not very difficult, not at all difficult). Baseline demographic information was also self-reported, including primary language, number of children and dependents, number of caregivers in the household, occupation, insurance status, working status, zip code, age, and race/ethnicity. There was an opportunity for open-ended responses at the conclusion of the survey.

Study data was collected and managed through REDCap electronic data capture tools hosted by UT Southwestern and analyzed in Excel and Stata 16. ​ Demographics, categorical survey responses, and Likert scale responses were reported with frequencies and summary statistics. Open-ended responses were reviewed and collated by salient topics.

## Results

The survey response rate was 96.7% among 336 respondents. Table 1 represents our study population with baseline characteristics. Participants were similar in sociodemographic characteristics as our health system population [35]. The mean age of survey respondents was 31.3 ± 7.9, and 213 (72.2%) identified as Hispanic and 60 (20.3%) identified as non-Hispanic Black or African-American. Approximately half (54.3%) were not currently working or pursuing studies, though 22.0% were employed full-time. While the majority of patients preferred English as their primary language, 37.6% preferred Spanish. Most individuals (61.3%) reported a household size of 1–4 people, while 38.0% reported 5–8 people in the home. Most respondents (59.3%) reported being the only caregiver in the home, though 34.4% reported an additional caregiver. The majority of respondents (70.6%) reported 1–2 dependents, and 25.5% reported 3–4 dependents. Approximately half of respondents (50.9%) declined having a regular provider for their healthcare; 34.5% reported seeing their obstetrician/gynecologist (OB/GYN) as their usual source of care, compared to 39.9% reporting seeking primary care.

**Table 1:** Baseline characteristics of survey respondents sampled in childcare needs assessment

*N* = 336	Number of patients (%)
**Mean age** **±** **SD (years)**	31.3 *±* 7.9
**Race/ethnicity** Hispanic or Latino Non-Hispanic White Non-Hispanic Black/African American Other	213 (72.2) 16 (5.4) 60 (20.3) 6 (2.0)
**Work status** Not working or a student and not looking for work Part-time work Full-time work Self-employed Looking for work Full-time student	158 (54.3) 41 (14.1) 64 (22.0) 4 (1.4) 13 (4.5) 11 (13.8)
**Language** English Spanish	214 (62.4) 129 (37.6)
**Number of people in home** 1–4 5–8 9+	205 (61.3) 127 (38.0) 3 (0.9)
**Number of caregivers in home** 1 (respondent) 2 3 4	198 (59.3) 115 (34.4) 18 (5.3) 3 (0.9)
**Number of dependents in home** 1–2 3–4 5–6 7+	236 (70.6) 85 (25.5) 12 (3.6) 1 (0.3)
Health insurance* Yes No	168 (50.9) 162 (49.1)
**Regular doctor or place to receive healthcare** Yes No	161 (49.1) 167 (50.9)
**Usual source of care** Primary care doctor Obstetrician gynecologist Other specialist Unknown	131 (39.9) 113 (34.5) 36 (11.0) 66 (20.1)
**Usual setting for routine visits** Community clinic Hospital-based specialty clinic Family planning clinic Doctor’s office Other place Don’t go to routine visits	109 (33.3) 85 (26.0) 20 (6.1) 42 (12.8) 10 (3.0) 59 (18.0)
**Usual setting for sick visits** Emergency room Hospital-based specialty clinic Doctor’s office Community clinic Urgent care clinic Walk-in clinic Don’t go to sick visits	128 (39.0) 113 (34.5) 10 (3.0) 35 (10.7) 9 (2.7) 9 (2.7) 23 (7.0)
**Transportation to appointment day of interview** Car Someone else dropped me off Bus Train Rideshare/taxi	235 (70.9) 56 (17.3) 16 (5.0) 5 (1.5) 11 (3.4)
**Type of care seeking day of interview** Obstetrics and Gynecology Medical and Surgical Oncology Community-based Primary Care Hospital-based Specialty Care Diagnostic Encounters	58 (17.8) 147 (45.0) 63 (19.3) 29 (8.9) 29 (8.9)

*Respondents often perceive Parkland Financial Assistance (county-funded charity coverage) as a form of insurance

**Fig. 1:**
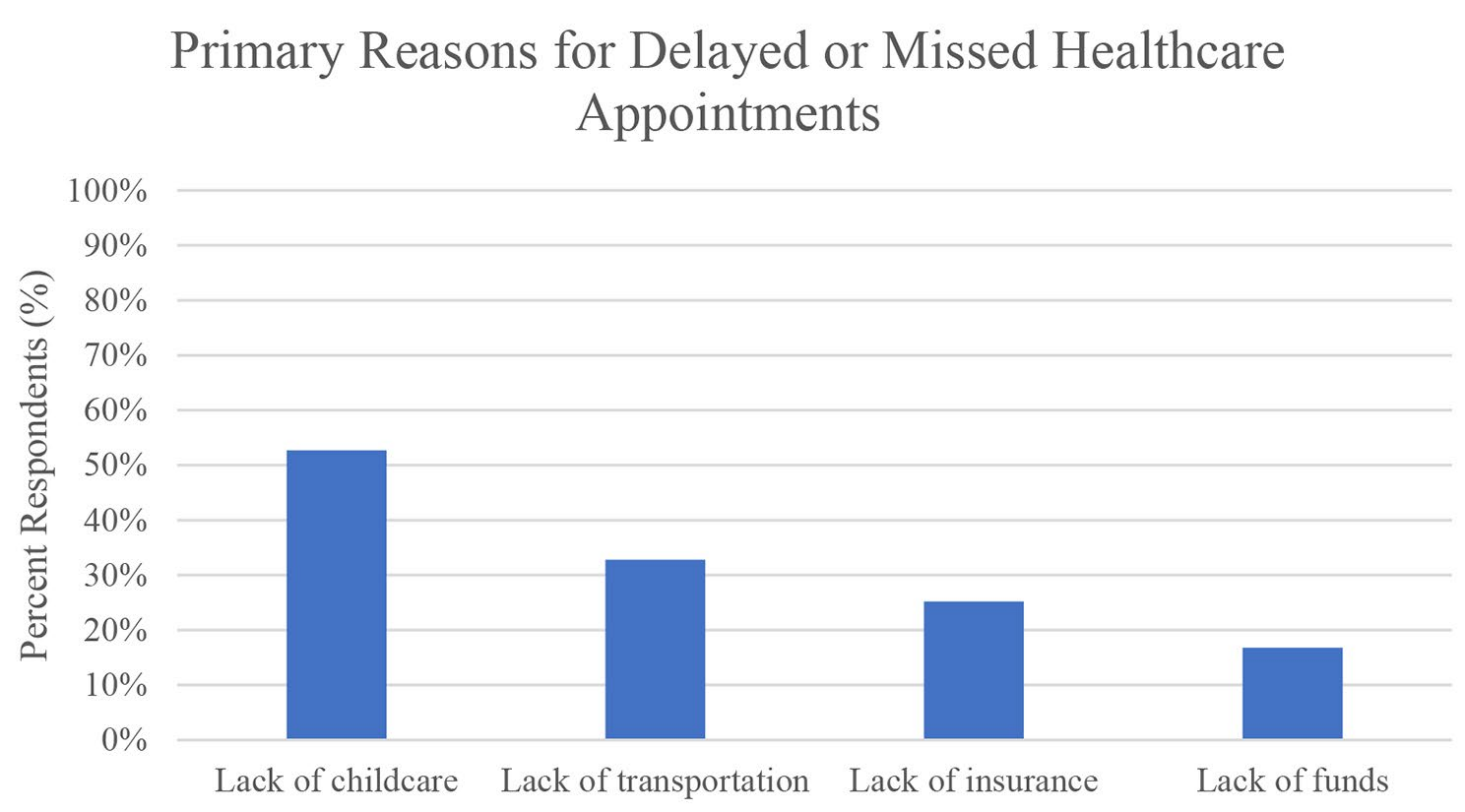
*N* = 121, respondents who reported delaying or missing a healthcare appointment in the past 12 months

Mean daily number of patients in waiting rooms was 25.0 ± 14.0, and mean daily number of children in waiting rooms was 5.2 ± 3.5. 121 (36.0%) reported delaying healthcare at least once in the past 12 months, and among this group, 54.5% reported missing an appointment due to lack of childcare (Fig. 1). On average, those 121 respondents delayed 3.7 ± 2.7 appointments per year. When asked about the length of delayed care, 30.9% reported delaying between 1 week to 1 month, and 38.2% reported delaying care further, between 1 and 6 months. Visits most frequently missed were check-ups and well visits (86.8%), however 31.8% reported missing problem visits including specialty appointments and oncologic care. Lack of childcare was the most frequently cited reason for missing care followed by lack of transportation and lack of insurance (Fig. 1). White (68.8%) and Black (55.0%) respondents were more likely to report delaying care in the past 12 months due to childcare needs in comparison with Hispanic respondents (34.3%).

**Fig. 2:**
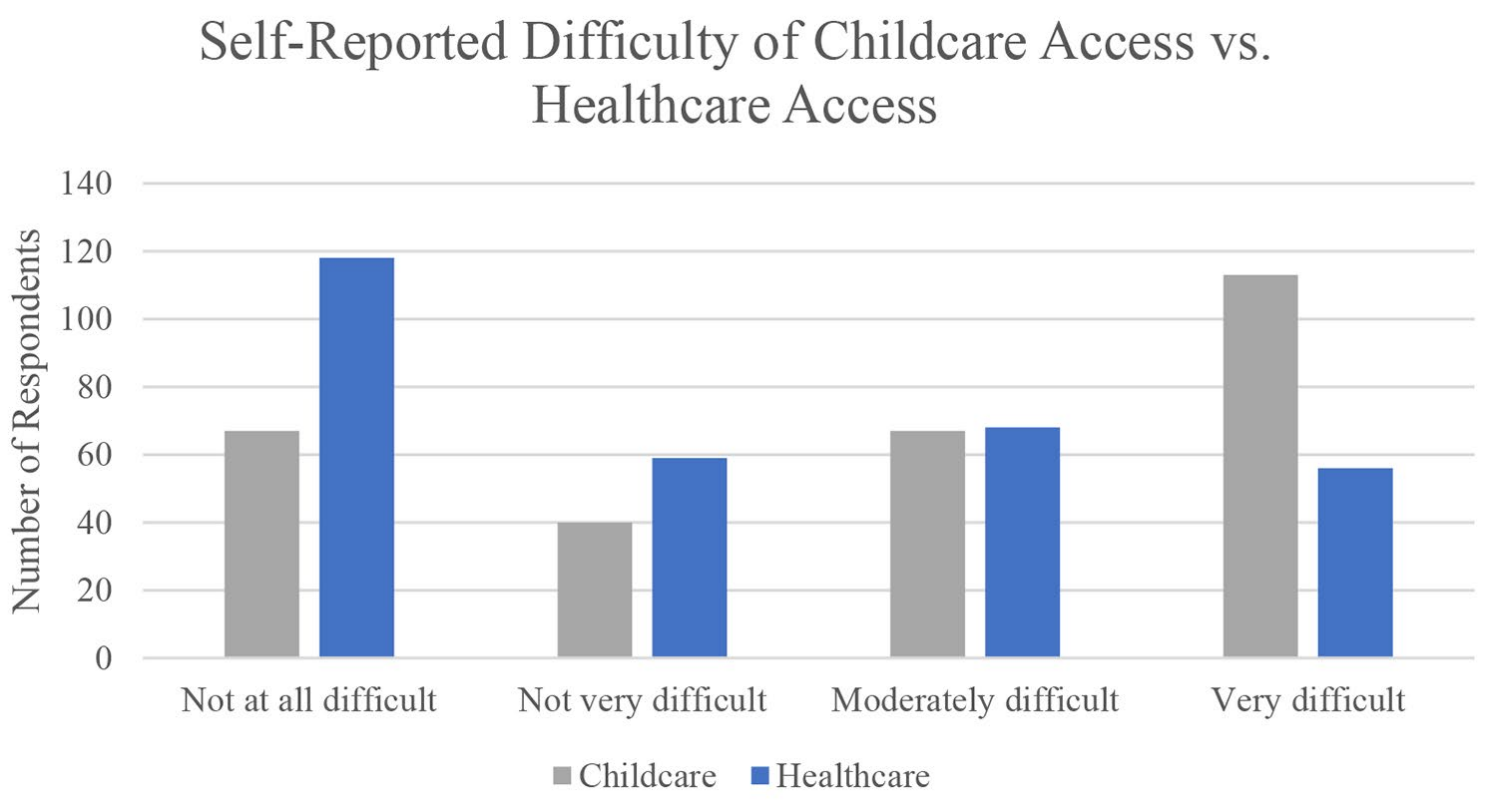
Childcare vs. healthcare access, *p* < 0.001

Most women (84.3%) reported being responsible for all or almost all their childcare needs. Figure 2 demonstrates increased self-reported difficulty in accessing childcare and compared to difficulty in accessing healthcare. When asked about experiences balancing healthcare and childcare needs, 69.1% of women reported bringing their child to a healthcare appointment due to lack of childcare, of whom 74.7% reported bringing them into the exam room or leaving them in the waiting room with someone (27.5%). Women shared their approaches managing childcare needs during scheduled healthcare appointments (Fig. 3). Moreover, 40.6% had left a healthcare appointment early to pick up their children, left before seeing the doctor (56.0%) or before the appointment was complete (42.4%). A subgroup (41.3%) specifically reported delaying or missing healthcare because they were not able to schedule an appointment when their child was in school.

**Figure:**
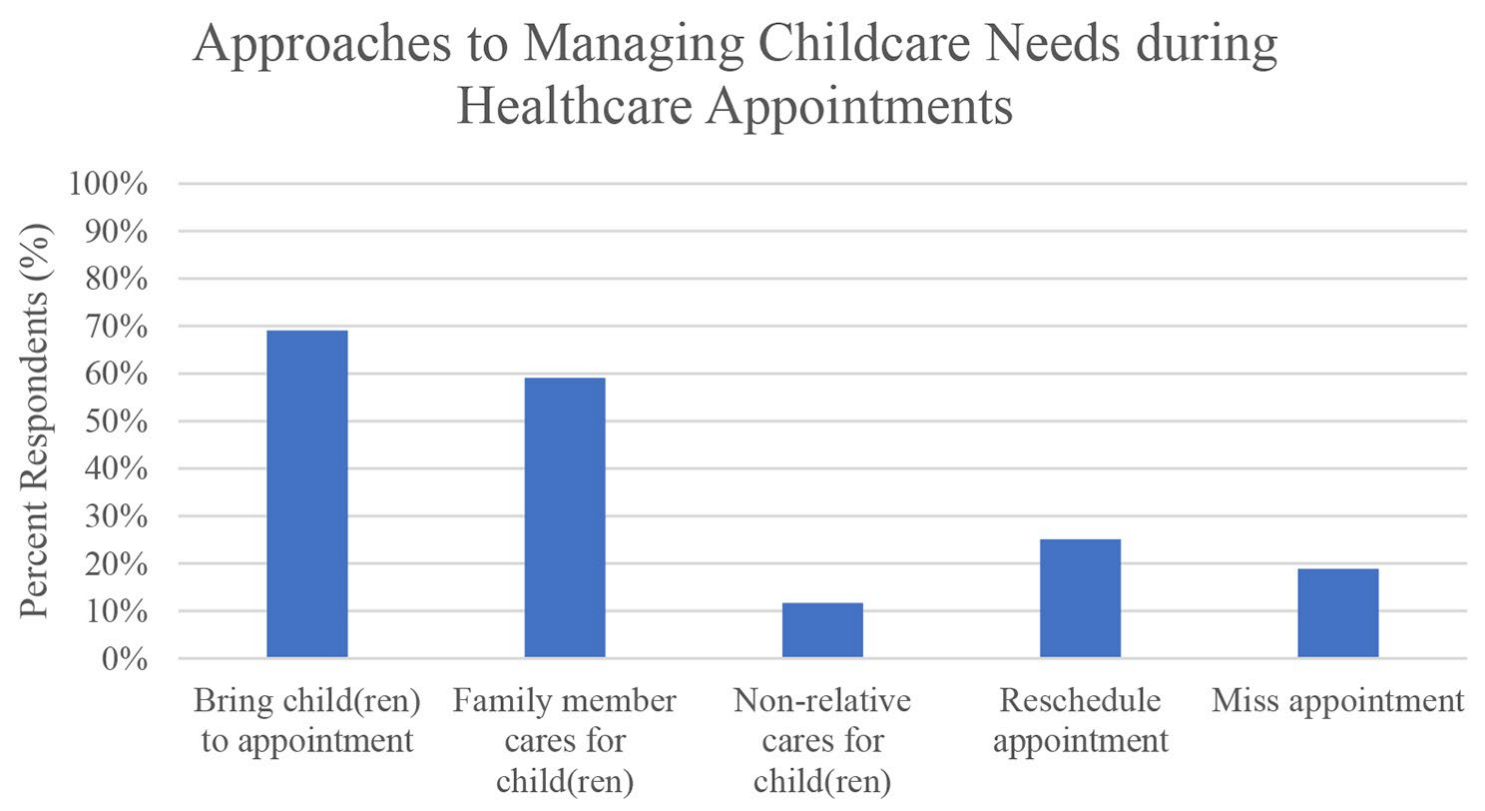
Figure 3

Only 22.3% had ever enrolled their children in a childcare center, and most common reasons for not using one included alternative childcare means with relative/nonrelative caretakers (65.8%). Others reported concerns about safety (25.8%) and lack of affordability (19.6%). Of the 64 women with daycare experience, 68.7% reported overall satisfaction with their experience.

The majority of those surveyed (87.9%) affirmed that a hospital-based daycare would help them attend their healthcare visits. Those unsure most commonly said they would use it in an emergency, and those who declined interest in daycare mainly cited lack of necessity as their current methods of childcare were sufficient. 48.2% reported needing 3–4 h for a single healthcare visit, and 46.5% were willing to pay $11–20 for this amount of childcare. Women ranked the following as extremely important interventions in a childcare center: opportunities to meet staff (81.0%), healthy eating education (80.0%), hand washing education (76.7%), structured activities (75.9%), snacks (66.8%), healthcare checkups for children (64.6%), social support for families (63.2%), and tours of the center (59.5%).

Open-ended answers revealed patterns describing women’s shared experiences managing their own healthcare needs with childcare responsibilities (Table 2). Common topics included difficulty balancing childcare needs with health system policies of clinical spaces, concerns about engaging with daycare centers, interest in hospital-based childcare services, and needs and desires in a potential childcare center.

**Table 2:** Salient topics recurring in open-ended survey responses

Topic	Aggregate Free Responses
Childcare barriers to healthcare access	“It is already so hard to get an appointment, but finding a babysitter is even harder.” “Childcare has been my biggest barrier to attending appointments, especially after my first delivery.” “I had to call a friend to pick up my son today because the staff would not let him into the exam room with me. I took off work to come today and would have had to reschedule if she couldn’t get him.” “My friend is watching my children in the elevator bay because they aren’t allowed in the waiting room right now.” “I have had to miss chemo appointments because of childcare issues, and kids are not allowed at these appointments.” “I sometimes pull my kids out of school to bring them to my appointment.” “I planned my whole day around this appointment.” A respondent asks the interviewer to stay with her infant in the waiting room
Concerns about a childcare center	“My son is disabled and I worry that they would not be able to care for him and kids with special needs.” “When I had my kids in daycare, the staff did not do as good of a job as they could probably because they themselves were not happy.” “It’s not hard to find a daycare center, it’s hard to find one that is good and affordable.” “I’ve heard too many bad stories on the news.” “I don’t trust daycare” “I stopped using a babysitter because [it was] too expensive” “There should be background checks and an open-door policy.” “Cameras with live streaming would give me more peace of mind.” “You could give the kids and parents wristbands like at church daycares.” “I would only use in an emergency situation or no other option.” “Good ratio of kids to caretakers is necessary” “Cleanliness and safety” “Separate the sick and healthy kids”
Interest in hospital-integrated childcare	“This would be so useful as I don’t have family in the area!” “Glad y’all thought about this.” “It would be wonderful to have a place where my children could wait for a relative to pick them up if I need to be admitted from an appointment.” “Moms need to be able to take care of themselves so they can take care of their families.” “Sooner the better. I’m interested in working in [hospital-based daycare].” “I would trust it because it’s right next to the hospital.” “I appreciate the survey and am looking forward to a daycare.” “Good thing that will benefit moms, especially single moms and moms with working spouses” “I think this idea would be great because I see all the dads taking care of children or trying to in waiting rooms. I also think this would have helped me a lot when I was raising kids.” “Although TV has decreased my trust in centers, I would trust a center at Parkland”
Preferences for services included in a childcare center	“There should be things for older kids to do too. Especially video games. Teenagers will go anywhere there’s FIFA.” “Give the kids free balloons and snacks.” “Shuttle bus would be good” “I think education is one of the most important things they can do for kids” “I would like to get to know place first and meet caretakers” “Thorough background check and cameras” “You should have crafts and games for the younger kids.” “I have experience with at home and public daycare and recommend consideration of allergies, age-appropriate activities, special needs, wall with pictures and descriptions of care providers, bundling childcare into PFA [Parkland Financial Assistance].”

## Discussion

This cross-sectional survey of women seeking care in a large, urban health system found that more than half of women who reported missing or delaying care in the past year attributed childcare needs as the primary barrier to accessing healthcare. In addition, on average respondents rated access to childcare as more difficult than access to healthcare. Current approaches to navigating competing childcare needs during healthcare appointments included relying on social networks and bringing children to healthcare settings for appointments. Few patients in our sample had prior experience with daycare centers, but survey responses reveal patient interest in hospital-based childcare services. These key findings supports previous findings from the Commonwealth Fund [9] and the KFF Women’s Health Survey [10] by demonstrating the burden of childcare barriers faced by women in underserved settings. Our needs assessment confirmed that childcare is a significant barrier to accessing healthcare, affecting women of all races and ethnicities in our county-funded safety-net health system.

This is one of the first studies to capture the prevalence of childcare as a barrier to healthcare access within a health system and describe in detail women’s unique experiences navigating competing healthcare and childcare needs. We surveyed a diverse cross-section of clinics representative of where women with childcare responsibilities seek healthcare, and thus may experience difficulties balancing these responsibilities with their own healthcare needs. Of note, our health system prohibits children younger than 13 unattended in clinical spaces, though variably implements these guidelines in the ambulatory clinics. There were unifying patterns amongst survey participant open-ended responses, specifically that affordable, convenient, and high-quality childcare is a need among our patient population and that safety and trust are top priorities when considering childcare. In addition, caretakers surveyed ranked health checkups and nutritional counseling for children, opportunities to meet staff, and extended social support for families as important factors in their decision to use childcare services. These secondary interventions may be introduced in the future in integrated and family-centered childcare centers, including through current interventions to deliver health system-integrated childcare services [36]. 

Our needs assessment suggested that white and Black women were more likely to delay healthcare in the past 12 months due to lack of childcare compared to Hispanic women. These differences may be attributable to increased collective family engagement in childcare responsibilities and cultural beliefs regarding childcare responsibilities among the Hispanic community [37, 38]. Previous social science research has demonstrated differences in the role of social support networks in childcare and family financial support across racial/ethnic groups [39–41]. Our observations are consistent with prior research in other disciplines examining cultural differences in values around childcare within the environment of healthcare delivery. These findings are integral for the design of culturally sensitive, patient-centered interventions to address childcare as a barrier to healthcare access. Additional research is needed to understand the cultural differences and intersectionality [42] that shape childcare barriers to healthcare.

Results should be interpreted within the context of our study limitations. This was a cross-sectional analysis of a pragmatic needs assessment; this survey was not designed to test for associations between childcare needs and patient factors. The survey was conducted in a single, safety-net health system, which may impact the generalizability of these findings. Limitations include patient recall bias when self-reporting delays in healthcare and the factors that impacted their ability to attend appointments. Response bias, particularly when responding to childcare center utilization, may have also been present as the survey was directly administered by the researchers. Notably, this needs assessment was administered in 2019, prior to the COVID-19 pandemic. Since this survey was administered, we anticipate patients may have different childcare needs related to workforce changes after the pandemic [16], and enforcement of childcare policies in clinical spaces have intensified. These factors may affect the current experience of childcare barriers in our health system. Strengths of our study include the high response rate and large number of respondents, and the inclusion of information regarding anticipated use and factors that would impact daycare center utilization. Another significant strength is the diverse patient sample reflective of our underserved safety-net patient population, a representative population for examining SDOH like childcare. Further studies are needed to understand the heterogeneity in childcare barriers in other vulnerable populations and the interaction of childcare needs alongside other SDOH.

Our study findings highlight the need for childcare policy reform as not only an economic imperative, but as a public health need. The challenges of childcare access in the United States has been consistently framed in terms of parental employment [43, 44], however these preliminary findings reinforce the public health implications of lack of access to childcare. Though there has been executive action to decrease costs of caregiving [45], federal legislation to increase access to childcare for low-income families remains pending [46]. Current advocacy efforts to increase access to childcare through childcare subsidies, tax credits, and Head Start and Early Head Start programs have centered on the impact of childcare on childhood development and parental workforce engagement [44, 47], however strengthening these programs could furthermore have important implications for the health of caregivers of children. Our findings of childcare needs presenting a barrier to healthcare access could support future childcare policy efforts by underscoring the benefits of childcare reform on public health as well as the labor market. Furthermore, these findings could suggest an opportunity for childcare needs addressed through healthcare system payment models (e.g., Medicaid transportation assistance [48]). 

This study demonstrates that childcare is a commonly reported reason for delays in healthcare. While childcare needs may impact patients in our community more significantly than in others, the prevalence of the concern, regardless of race or work status, does support that childcare is a stressor which impacts healthcare access. In this population, the struggle to balance competing needs of one’s own healthcare and childcare responsibilities often pit these two needs directly against one another. What we found was sobering, but not unexpected. Women deprioritize their own healthcare to the more pressing need to provide childcare and reported that accessing childcare was more difficult than accessing healthcare. Future research is needed to investigate interventions to balance these competing needs, potentially through integrating childcare support in the healthcare setting.

### Electronic supplementary material

Below is the link to the electronic supplementary material.

Supplementary Material 1

## Data Availability

The datasets generated and/or analysed during the current study are not publicly available due to patient privacy policies of our health system but are available from the corresponding author on reasonable request.
