# Correction: Childcare needs as a barrier to healthcare among women in a safety-net health system

**DOI:** 10.1186/s12889-024-19230-1

**Published:** 2024-07-05

**Authors:** Priyanka Gaur, Anisha P. Ganguly, Madyson Kuo, Robert Martin, Kristin S. Alvarez, Kavita P. Bhavan, Kimberly A. Kho

**Affiliations:** 1 Department of Gynecology and Obstetrics, The Johns Hopkins University School of Medicine, Baltimore, MD United States of America; 2 Center of Innovation and Value at Parkland, Parkland Health, Dallas, TX United States of America; 3 Department of Internal Medicine, University of Texas Southwestern Medical Center, Dallas, TX United States of America; 4 Parkland Health Center for Innovation and Value at Parkland, 5200 Harry Hines Blvd, Dallas, TX 75235 USA; 5 Department of Obstetrics and Gynecology, Methodist Health System, Dallas, TX United States of America; 6 Department of Obstetrics and Gynecology, University of Texas Southwestern Medical Center, Dallas, TX United States of America

**BMC Public Health (2024) 24:1608.**

10.1186/s12889-024-19125-1.

In the original publication of this article Table 3 was a duplicate of Table 2. The original article has been corrected to remove the duplicate. The publisher apologizes for the inconvenience caused.

