# Supplementary material for: Childcare needs as a barrier to healthcare among women in a safety-net health system
Source: BMC Public Health. 2024 Jun 17;24:1608. doi: 10.1186/s12889-024-19125-1 (PMC11181530; doi:10.1186/s12889-024-19125-1)
Supplement: Supplementary file 1 — Supplementary Material 1 [file 12889_2024_19125_MOESM1_ESM.docx]

Hello, my name is <research assistant> with Parkland Health & Hospital System. We are conducting a survey to find out more about the health care and child care needs of patients in our clinics. We would like to ask you a few questions while you are waiting for your appointment. May I continue?

Would you like to continue in English or Spanish?

Thank you!

Do you have any children living at home under the age Yes of 13? No

Do you have or share child care responsibilities for Yes

the children under age 13 in the home? No


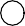

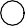

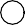

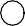


Time now

Language English

Spanish


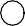

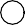


Name of clinic Maple

Lake West Hatcher


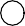

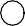

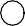

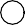

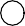

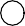

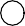

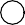

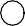

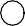

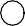

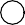

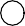

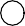

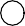

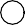

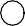


deHaro-Saldivar Obstetrics Gynecology Family Medicine Southeast

Internal Medicine Breast Imaging Colonoscopy Breast Oncology Gynecology

Gynecology Oncology Ron J. Anderson Medical Oncology Surgical Oncology Other

Please specify.

Appointment date

**You and your family**

What is your relationship to the children under age Parent/Stepparent


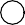

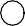

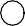

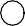

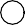


13 living in your home? Grandparent

Foster parent Other relative Other non-relative

How many people live with you in your home, including 1


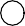

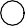

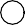

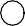

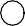

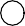

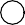

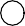

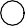

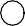


you? 2

3

4

5

6

7

8

9

10+

How many of the children living in your home are 5 0

years of age or younger? 1


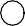

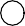

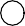

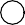

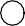

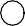

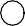

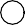

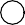

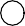

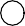


2

3

4

5

6

7

8

9

10+

How many of the children living in your home are 6-13 0

years of age? 1


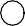

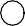

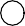

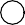

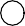

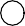

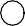

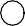

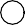

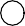

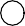


2

3

4

5

6

7

8

9

10+

How many of the children living in your home are 0

14-17 years of age? 1


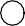

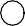

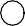

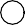

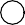

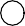

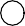

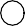

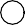

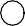

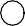


2

3

4

5

6

7

8

9

10+

How many people living in your home are 18 years of 1


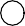

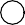

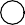

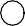

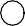

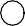

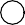

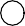

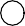

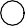

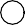


age or older, including you and any children? 2

3

4

5

6

7

8

9

10+

10+

How many of these people in your home 18 years of age 1


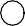

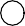

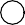

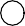

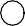

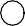

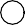

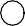

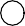

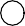


or older help with child care, including you? 2

3

4

5

6

7

8

9

10+

For how many people are you their primary caregiver? 1


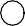

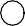

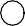

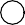

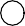

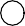

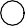

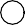


2

3

4

5

6

7

8

9

10+

**Your health**

In general, how would you rate your overall health? Excellent Very good Good

Fair Poor

Do you have any type of health insurance right now? Yes No

Do you have a regular doctor or place that you go to Yes

when you are sick or for a checkup? No

Who usually provides your health care? Primary care doctor

Obstetrician gynecologist Other specialist

Unknown

Please specify.

What type of place do you usually go to for routine Doctor's office

health care visits when nothing is wrong? Hospital-based specialty clinic Community or public health clinic Family planning clinic

Walk-in clinic

School or university clinic Urgent care clinic Emergency room

Don't go to routine health care visits

Do you go to Parkland for routine health care visits Yes

when nothing is wrong? No

Where do you usually go for routine health care visits when nothing is wrong?

What type of place do you go to usually when you are Doctor's office

sick? Hospital-based specialty clinic

Community or public health clinic Family planning clinic

Walk-in clinic

School or university clinic Urgent care clinic Emergency room

Don't go to sick visits

Do you go to Parkland when you are sick? Yes No

Where do you usually go when you are sick?

What type of care do you go to Parkland for? Check Well visits, checkups, annual exams all that apply. Prenatal care or family planning

Medical test or treatment Surgery or procedure

Pick up medications Emergency care

Sick visits

**Your health care**

Currently, how difficult is it for you to get medical Very difficult

care when you need it? Moderately difficult

Not very difficult Not at all difficult

How did you get to your appointment today? Bus Train Car

Someone else dropped you off Ride share/taxi

Walking

In the past 12 months, was there a time you delayed Yes

or missed health care you needed? No

In the past 12 months, have you delayed or missed You could not afford it

health care you needed for any of the following You didn't have health insurance

reasons? Check all that apply. You didn't have child care

You didn't have transportation

You could not take time off from work/school You could not find a regular doctor

You could not find a doctor who accepts your insurance plan

The doctor's office/clinic was not open when you could get there

The wait time for an appointment was too long in the doctor's office/clinic

The wait time once you got to the doctor's office/clinic was too long

How many times have you delayed or missed an 1

appointment in the past 12 months because you could 2

not afford it? 3

4

5

6

7

8

9

10+

Generally, how long did you delay your care, or how One week or less

long did it take to reschedule your appointment More than a week, but less than 1 month because you could not afford it? 1 month to 6 months

More than 6 months to 12 months Unknown

How many times have you delayed or missed an 1

appointment in the past 12 months because you didn't 2

have insurance? 3

4

5

6

7

8

9

10+

Generally, how long did you delay your care, or how One week or less

long did it take to reschedule your appointment More than a week, but less than 1 month because you didn't have insurance? 1 month to 6 months

More than 6 months to 12 months Unknown

How many times have you delayed or missed an 1

appointment in the past 12 months because you didn't 2

have child care? 3

4

5

6

7

8

9

10+

Generally, how long did you delay your care, or how One week or less

long did it take to reschedule your appointment More than a week, but less than 1 month because you didn't have child care? 1 month to 6 months

More than 6 months to 12 months Unknown

How many times have you delayed or missed an 1

appointment in the past 12 months because you didn't 2

have transportation? 3

4

5

6

7

8

9

10+

Generally, how long did you delay your care, or how One week or less

long did it take to reschedule your appointment More than a week, but less than 1 month because you didn't have transportation? 1 month to 6 months

More than 6 months to 12 months Unknown

How many times have you delayed or missed an 1

appointment in the past 12 months because you could 2

not take time off from work/school? 3

4

5

6

7

8

9

10+

Generally, how long did you delay your care, or how One week or less

long did it take to reschedule your appointment More than a week, but less than 1 month because you could not take time off from work/school? 1 month to 6 months

More than 6 months to 12 months Unknown

How many times have you delayed or missed an 1

appointment in the past 12 months because you could 2

not find a regular doctor or clinic? 3

4

5

6

7

8

9

10+

Generally, how long did you delay your care, or how One week or less

long did it take to reschedule your appointment More than a week, but less than 1 month because you could not find a regular doctor or clinic? 1 month to 6 months

More than 6 months to 12 months Unknown

How many times have you delayed or missed an 1

appointment in the past 12 months because you could 2

not find a doctor who accepts your insurance plan? 3

4

5

6

7

8

9

10+

Generally, how long did you delay your care, or how One week or less

long did it take to reschedule your appointment More than a week, but less than 1 month because you could not find a doctor who accepts your 1 month to 6 months

insurance plan? More than 6 months to 12 months

Unknown

How many times have you delayed or missed an 1

appointment in the past 12 months because the 2

doctor's office/clinic was not open when you could 3

get there? 4

5

6

7

8

9

10+

Generally, how long did you delay your care, or how One week or less

long did it take to reschedule your appointment More than a week, but less than 1 month because the doctor's office/clinic was not open when 1 month to 6 months

you could get there? More than 6 months to 12 months Unknown

How many times have you delayed or missed an 1

appointment in the past 12 months because the wait 2

time for an appointment was too long in the doctor's 3

office/clinic? 4

5

6

7

8

9

10+

Generally, how long did you delay your care, or how One week or less

long did it take to reschedule your appointment More than a week, but less than 1 month because the wait time for an appointment was too 1 month to 6 months

long in the doctor's office/clinic? More than 6 months to 12 months Unknown

How many times have you delayed or missed an 1

appointment in the past 12 months because the wait 2

time once you got to the doctor's office/clinic was 3

too long? 4

5

6

7

8

9

10+

Generally, how long did you delay your care, or how One week or less

long did it take to reschedule your appointment More than a week, but less than 1 month because the wait time once you got to the doctor's 1 month to 6 months

office/clinic was too long? More than 6 months to 12 months Unknown

Please tell us the one main reason you delayed or You could not afford it

missed health care you needed in the past 12 months. You didn't have health insurance

You didn't have child care

You didn't have transportation

You could not take time off from work/school You could find a regular doctor

You could not find a doctor who accepts your insurance plan

Doctor's office/clinic was not open when you could get there

The wait time for an appointment was too long in the doctor's office/clinic

The wait time once you got to the doctor's office/clinic was too long

In the past 12 months, have you delayed or missed any Well visits, checkups, annual exams including pap of the following? Check all that apply. tests, colonoscopies, and mammograms

Medical test or treatment Procedure or surgery

Filling a prescription Dental care

Eye care like eye glasses

How many times have you delayed or missed a well 1

visit, checkup, or annual exam in the past 12 months? 2

3

4

5

6

7

8

9

10+

Generally how long did you delay your well visit, One week or less

checkup, annual exam, or how long did it take to More than a week, but less than 1 month reschedule it? 1 month to 6 months

More than 6 months to 12 months Unknown

How many times have you delayed or missed a medical 1

test or treatment in the past 12 months? 2

3

4

5

6

7

8

9

10+

Generally how long did you delay your medical test or One week or less

treatment, or how long did it take to reschedule it? More than a week, but less than 1 month

1 month to 6 months

More than 6 months to 12 months Unknown

How many times have you delayed or missed a procedure 1

or surgery in the past 12 months? 2

3

4

5

6

7

8

9

10+

Generally how long did you delay your procedure or One week or less

surgery, or how long did it take to reschedule it? More than a week, but less than 1 month

1 month to 6 months

More than 6 months to 12 months Unknown

How many times have you delayed or missed filling a 1

prescription in the past 12 months? 2

3

4

5

6

7

8

9

10+

Generally, how long did you delay filling your One week or less

prescription, or how long did it take to refill it? More than a week, but less than 1 month 1 month to 6 months

More than 6 months to 12 months Unknown

How many times have you delayed or missed dental care 1

in the past 12 months? 2

3

4

5

6

7

8

9

10+

Generally, how long did you delay or miss your dental One week or less

care, or how long did it take to reschedule it? More than a week, but less than 1 month 1 month to 6 months

More than 6 months to 12 months Unknown

How many times have you delayed or missed eye care in 1

the past 12 months? 2

3

4

5

6

7

8

9

10+

Generally, how long did you delay or miss your eye One week or less

care, or how long did it take to reschedule it? More than a week, but less than 1 month 1 month to 6 months

More than 6 months to 12 months Unknown

**Your child care**

Who in your family usually takes care of your You

children's needs? Check all that apply. Immediate family Extended family Friend

Professional child care

What is their relationship to you? Check all that Spouse

apply. Parent/Stepparent

Parent-in-law Sibling

Sibling-in-law Other relative

Please specify.

About how much of child care do you do? All or almost all More than half About half

Less than half Almost none

Who do you prefer to take care of your children if Immediate family

you cannot? Check all that apply. Extended family Friend

Professional child care

What is their relationship to you? Spouse

Parent/Stepparent Parent-in-law

Sibling

Sibling-in-law Other relative

Please specify.

When do you prefer to schedule your health care Mornings

appointments? Afternoons

Evenings

No preference

Why do you prefer to schedule your health care Children are in school

appointments at this time? Easier for work schedule Easier for household schedule Other personal preference

What do you do when you have to take care of your Bring children to appointment child and have a health care appointment? Check all Find a relative babysitter

that apply. Find a non-relative babysitter

Reschedule appointment Miss appointment

Do you usually pay for the babysitter? Yes No

How much do you usually pay for the babysitter?

How difficult is it for you to find someone to take Very difficult

care of your children when you have a health care Moderately difficult

appointment? Not very difficult

Not at all difficult

Have you ever had to bring your children to a health Yes

care appointment? No

What do you usually do with your children when it is Leave them in the waiting room with someone you time to see the doctor? Check all that apply. know

Leave them in the waiting room with someone you don't know

Leave them in the waiting room alone Bring them into the exam room

Have you ever had to leave a health care appointment Yes early to pick up your children? No

When did you leave your health care appointment? Left before seeing the doctor

Check all that apply. Left before appointment was completed

Left before picking up medications or supplies Left before getting labs

Do you currently have children in preschool through Yes

high school? No

What grades are your children in? Check all that Preschool

apply. Kindergarten-5th grade

6th-8th grade 9th-12 grade

Have you delayed or missed a health care appointments Yes because you could not schedule an appointment when No your child was in school?

How many times have you delayed or missed a health 1

care appointment in the past 12 months because you 2

could not schedule an appointment when your child 3

was in school? 4

5

6

7

8

9

10+

Generally how long did you delay your care, or how One week or less

long did it take to reschedule your appointment? More than a week, but less than 1 month

1 month to 6 months

More than 6 months to 12 months Unknown

**Your thoughts on day care**

Have you ever enrolled your child in a day care Yes

center? No

What was your level of trust in the day care center Very poor

to take care of your children? Poor

Fair Good

Very good

What was your level of overall satisfaction with the Very poor

day care center? Poor

Fair Good

Very good

What are your reasons for not using a day care You did not need day care

center? Check all that apply. You never considered a day care You could not afford day care

You heard bad things about day care

You worried about your children's safety in day care

You worried about your children getting sick in day care

You were concerned about the day care's ability to tend to your children's specific needs

**For the following questions, please tell me whether you completely disagree, somewhat**

**disagree, neither disagree or agree, somewhat agree, or completely agree with each statement.**

Completely

disagree

Somewhat

disagree

Neither disagree

or agree

Somewhat agree Completely

agree

Day care staff show warmth and care when interacting with children.

Children receive the attention they need in day care centers.

Day care center staff receive appropriate training to care for children.

I feel my child will be safe when I leave them in a day care center.

Day care center equipment and toys are safe and maintained well.

It is easy for me to find a day

care center that is near me.

I know how to enroll my child in a day care center.

I would be able to meet the requirements to enroll my child in a day care like providing my child's immunization records.

Day care centers are an affordable option for child care.

I would be willing to pay for professional day care.

I prefer to pay a babysitter that comes to my home when I need help with child care instead of using a day care.

I usually do not pay for child care because I have relatives or non-relatives that help me.

| **A day care center for patients** |  | | | |
| --- | --- | --- | --- | --- |
| Would having a drop-off day care center for patients near Parkland hospital be helpful for you? | Yes No Maybe |  |  |  |
| Generally, how many hours of child care do you think | < 1 | 1-2 | 3-4 |  |
| you would need for a single health care appointment? | 5-6 | 7-8 | 9-10 |  |
|  | 11-12 | 13-14 |  | 15+ |
| How much would you be willing to pay or donate for $0 $1-10 $11-20 | | | | |
| this many hours of child care? | $21-30 | $31-40 | | $41-50 |
|  | $51-60 | $61-70 | | $71-80 |
|  | $81-90 | $91-100 | |  |
|  | $101+ |  | |  |

**How important would the following be in your decision to use a day care center near Parkland**

**while you are at your health care appointment?**

Health care checkups for cEhdiuldcraetnion on hand washing for children

Education on healthy eating for children

Structured activities for children Snacks for children

Social support for families

Tours of the day care center for families

Opportunities to meet the day care staff

Not at all

important

Slightly

important

Moderately

important

Very important Extremely

important

**More about you**

What is your age? 18 or younger

19

20

21

22

23

24

25

26

27

28

29

30

31

32

33

34

35

36

37

38

39

40

41

42

43

44

45

46

47

48

49

50 or older

How would you describe your race/ethnicity? White

Hispanic or Latino

Black or African American

Native American or American Indian Asian or Pacific Islander

Other

What is your zip code?

What is your current working status? Looking for work

Not working or a student and not looking for work Part-time working for pay at a job or business

Full-time working for pay at a job or business Self-employed

Full-time student Retired

How would you describe your work? Manager

Professional

Technician (process control, telecommunications) Clerical worker (secretary, clerk)

Service and sales worker (child care, teacher aide, salesperson, cashier, waiter, hairdresser)

Skilled agricultural, forestry, and fishery worker (farmer, crop and animal producer)

Craft and related trade worker (construction trades, garments, electronics, metal worker)

Plant and machine operator and assembler (textiles, chemicals, rubber, wood, drivers)

Laborer (cleaner, helper, food preparation, manufacturing)

Armed forces occupation

Interview notes

Time now
